# Design of Deep Eutectic Systems: Plastic Crystalline Materials as Constituents

**DOI:** 10.3390/molecules27196210

**Published:** 2022-09-21

**Authors:** Ahmad Alhadid, Sahar Nasrallah, Liudmila Mokrushina, Mirjana Minceva

**Affiliations:** 1 Biothermodynamics, TUM School of Life Sciences, Technical University of Munich (TUM), Maximus-von-Imhof-Forum 2, 85354 Freising, Germany; 2 Separation Science & Technology, Friedrich-Alexander-Universität Erlangen-Nürnberg (FAU), Egerlandstr. 3, 91058 Erlangen, Germany

**Keywords:** deep eutectic solvents, green solvents, solid–solid transition, solid–liquid equilibria, melting properties, differential scanning calorimetry

## Abstract

Deep eutectic solvents (DESs) are a class of green and tunable solvents that can be formed by mixing constituents having very low melting entropies and enthalpies. As types of materials that meet these requirements, plastic crystalline materials (PCs) with highly symmetrical and disordered crystal structures can be envisaged as promising DES constituents. In this work, three PCs, namely, neopentyl alcohol, pivalic acid, and neopentyl glycol, were studied as DES constituents. The solid–plastic transitions and melting properties of the pure PCs were studied using differential scanning calorimetry. The solid–liquid equilibrium phase diagrams of four eutectic systems containing the three PCs, i.e., L-menthol/neopentyl alcohol, L-menthol/pivalic acid, L-menthol/neopentyl glycol, and choline chloride/neopentyl glycol, were measured. Despite showing near-ideal behavior, the four studied eutectic systems exhibited depressions at the eutectic points, relative to the melting temperatures of the pure constituents, that were similar to or even larger than those of strongly nonideal eutectic systems. These findings highlight that a DES can be formed when PCs are used as constituents, even if the eutectic system is ideal.

## 1. Introduction

Deep eutectic solvents (DESs) are eutectic systems prepared by mixing two or more compounds to form a mixture with a melting temperature significantly lower than that of the individual constituents [1,2]. As a new generation of designer and green solvents, DESs are promising alternatives for overcoming the drawbacks of conventional solvents—particularly, their toxicity and environmental impacts [3,4,5]. Various DESs have been reported as potential green solvents that outperform conventional organic solvents in extraction, separation processes, and bioapplications [6,7,8,9,10,11,12].

For various applications, DESs must be liquid at the operating temperature. The solid–liquid equilibrium (SLE) phase diagram of DESs allows for identifying the melting temperature of the system at any composition. The SLE phase diagram and the position of the eutectic point depend on the nonideality of the liquid phase, the melting properties of the pure components, and the type of the formed solid phases [13].

In fact, the term DES is only applied for eutectic systems showing a substantial negative deviation from ideal behavior, i.e., strong hydrogen bonding interactions [14]. In many cases, mixing halide salts as hydrogen bond acceptors (HBAs) with carboxylic acids [15,16,17], polyols [18], sugars [19], sugar alcohols [20], or amides [21,22] as hydrogen bond donors (HBDs) results in the formation of eutectic systems with a substantial negative deviation from ideality and a low eutectic temperature. Accordingly, many studies regarding the SLE of DESs have focused on ionic constituents as HBAs. Nevertheless, the applicability of ionic DESs is somewhat restricted due to their high hygroscopicity, their thermal instability, and the toxicity of some halide salts [23]. Moreover, the strong electrostatic interactions occurring in the liquid phase endow ionic DESs with viscosity and a high density [24,25,26,27,28].

To overcome the main drawbacks of ionic DESs, particularly their toxicity and hygroscopicity, natural organic compounds have recently been suggested as constituents of hydrophobic nonionic eutectic systems [29,30,31,32]. Simple organic compounds have been used to prepare nonionic eutectic systems that were found to be superior to ionic DESs and ionic liquids in terms of viscosity, economy, and performance [33,34]. However, because most nonionic eutectic systems studied in the literature are nearly ideal mixtures, their eutectic temperature is not significantly lower than the melting temperatures of the pure constituents [6,35]. To construct eutectic systems with a large depression in the melting temperature at the eutectic point, mixing components that have low melting entropies and enthalpies is an effective strategy [36]. For instance, constituents with rigid and symmetrical molecular structures have been shown to possess sufficiently low melting entropies to form eutectic systems with low eutectic temperatures [35].

The present work studies plastic crystalline materials (PCs) as constituents of eutectic systems with low eutectic temperatures. PCs are solid compounds with disordered and symmetrical crystal structures, i.e., a cubic lattice, and thus they possess extremely low melting entropies and enthalpies. Due to their unique mechanical and conducting properties, PCs have been used in various applications [37,38]. In this study, three PCs that have the chemical nature of commonly used DES constituents, namely, monocarboxylic acids, alcohols, and diols, were investigated. The three PCs were mixed with L-menthol or choline chloride (ChCl) to form four eutectic systems. The solid–plastic transition and melting properties of the pure PCs and the SLE phase diagram of the four eutectic systems were measured using differential scanning calorimetry (DSC). The SLE phase diagram was modeled to obtain the position of the eutectic point of the system.

## 2. Results and Discussion

### 2.1. Properties of Pure PCs

**Figure 1:**
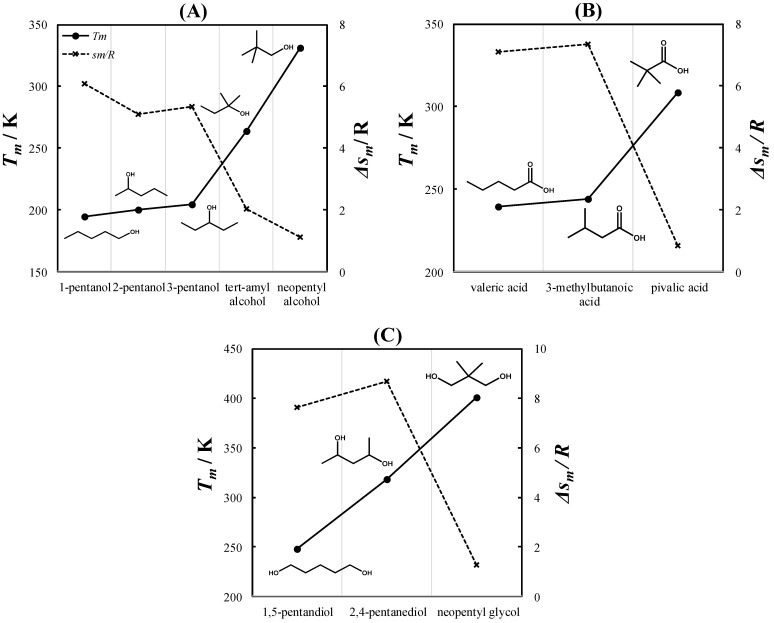
Melting temperatures (*T_m_*) and melting entropies (Δ*s_m_/R*) of various isomers of C5 alcohols (**A**), monocarboxylic acids (**B**), and diols (**C**). Experimental data were taken from Lohmann et al. [40] for 1-, 2-, and 3-pentanol; Parks et al. [41] for tert-amyl alcohol; Timmermans [42] for valeric and 3-methylbutanoic acid; Miller [43] for 1,5-pentanediol; and Mellan [44] for 2,4-pentanediol. The melting entropies of 3-methylbutanoic acid and 2,4-pentanediol are unavailable in the literature and were estimated using the method of Jain et al. [45].

Due to their symmetrical crystal structure, PCs exhibit an extremely low melting entropy. Timmermans [39] reported an upper limit of 5 kcal mol^−1^ K^−1^ (~2.5 R) for the melting entropies of PCs. As a result of their very small melting entropies, PCs tend to have higher melting temperatures than their chemical isomers. Figure 1 shows a comparison between the melting properties of various pentyl (C5) alcohol (Figure 1A), monocarboxylic acid (Figure 1B), and diol (Figure 1C) chemical isomers. As seen in Figure 1A, the melting temperatures of linear C5 alcohols (1-, 2-, and 3-pentanol) are similar. In contrast, tert-amyl and neopentyl alcohols exhibit significantly higher melting temperatures. Neopentyl alcohol is the only C5 alcohol that is solid at room temperature. Similarly, the melting temperature of pivalic acid (Figure 1B) and neopentyl glycol (Figure 1C) are significantly higher than those of their chemical isomers. The melting entropies of tert-amyl alcohol, neopentyl alcohol, pivalic acid, and neopentyl glycol are below Timmermans’s limit, indicating that these compounds are PCs.

**Figure 2:**
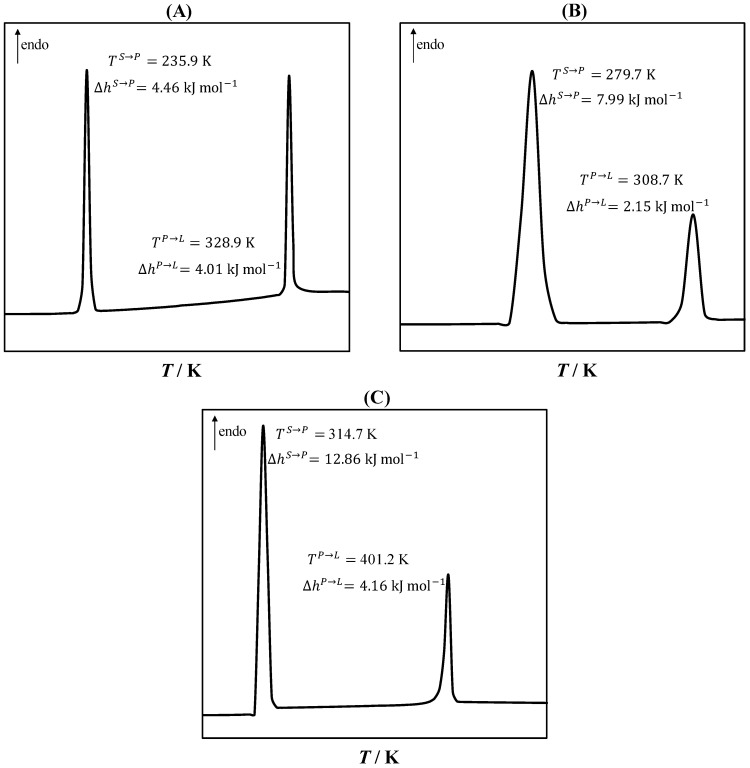
Differential scanning calorimetry curves of neopentyl alcohol (**A**), pivalic acid (**B**), and neopentylglycol (**C**), showing the solid–plastic transition temperature ($T^{S\to P}$) and enthalpy ($∆h^{S\to P}$) and the melting temperature ($T^{P\to L}$) and enthalpy ($∆h^{P\to L}$).

Upon cooling, PCs transform from high-symmetry disordered solid states (plastic states) to low-symmetry ordered solid states [37]. The solid–plastic transition can occur in a single or several phase transitions, and the associated enthalpy is larger than the plastic–liquid transition, i.e., the melting [39,46]. Figure 2 shows the DSC curves of the studied PCs, namely, neopentyl alcohol, pivalic acid, and neopentyl glycol. As seen in Figure 2, a single solid–plastic transition was observed. The solid–plastic transition (first peak) enthalpy was larger than the melting enthalpy (second peak). The largest difference between the solid–plastic transition and melting temperatures was observed in neopentyl alcohol, and the largest difference between the solid–plastic transition and melting enthalpies was found for neopentyl glycol.

### 2.2. Eutectic Systems with PCs

**Figure 3:**
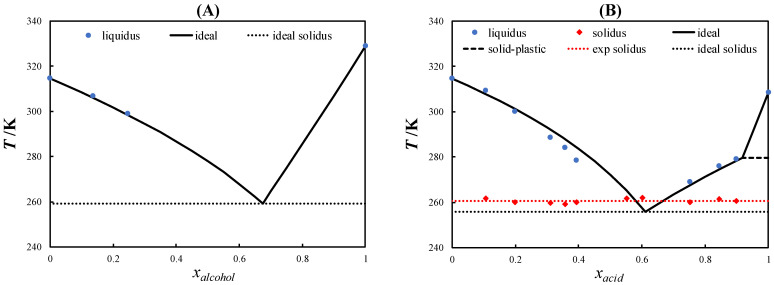

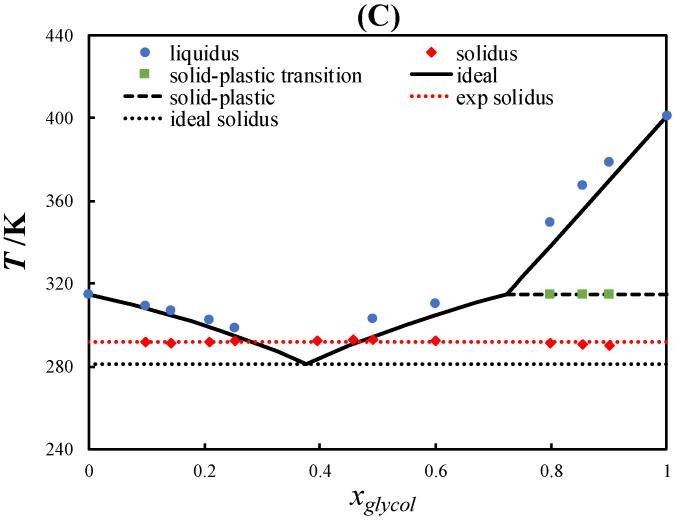
Solid–liquid phase diagram of L-menthol/neopentyl alcohol (**A**), L-menthol/pivalic acid (**B**), and L-menthol/neopentyl glycol (**C**).

In this work, the SLEs of three eutectic systems containing L-menthol and PCs were determined using DSC. Figure 3 shows the SLE phase diagram of L-menthol/neopentyl alcohol, L-menthol/pivalic acid, and L-menthol/neopentyl glycol.

No eutectic temperature for the L-menthol/neopentyl alcohol eutectic system (Figure 3A) could be measured due to the kinetic limitations in crystallization. The measured SLE data of the L-menthol liquidus line in the range of x_alcohol_ < 0.3 indicated that the system behaves ideally. Moreover, due to the similar chemical nature of L-menthol and neopentyl alcohol, i.e., both are alcohols, the liquid solution is expected to be ideal. Therefore, the eutectic temperature was estimated using the ideal solution model as 259.2 K. The solid–plastic transition of neopentyl alcohol did not influence the SLE phase diagram of the system because the solid–plastic transition temperature was lower than the eutectic temperature of the system.

As can be observed in the SLE phase diagram of the L-menthol/pivalic acid eutectic system shown in Figure 3B, the L-menthol liquidus line showed a slight negative deviation from the ideal behavior similar to that observed in L-menthol-based eutectic systems containing other monocarboxylic acids [35]. Meanwhile, the pivalic acid liquidus line showed a slightly positive deviation from the ideal behavior. Correspondingly, the eutectic temperature of the system was slightly higher than the ideal eutectic temperature.

Figure 3C displays the SLE phase diagram of the L-menthol/neopentyl glycol eutectic system, which exhibited a slightly positive deviation from the ideal behavior in the liquidus lines of L-menthol and neopentyl glycol. Accordingly, the eutectic temperature was higher than the ideal eutectic temperature. However, the difference between the eutectic temperature of the system and the melting temperature of neopentyl glycol was approximately 110 K, which is considerably high for a nearly ideal eutectic system.

As seen in Figure 3B,C, the course of the liquidus lines of pivalic acid and neopentyl glycol above the solid–plastic transition temperature was very steep due to the small melting enthalpies of the PCs. The slope of the liquidus line of the PCs decreased significantly below the solid–plastic transition temperature due to the relatively large solid–plastic transition enthalpy compared with the melting enthalpy. The order of the depression at the eutectic point relative to the melting temperature of the pure constituents was L-menthol/neopentyl alcohol > L-menthol/neopentyl glycol > L-menthol/pivalic acid, which was consistent with the difference between the solid–plastic transition and melting temperatures of the pure PCs (see Figure 2). Thus, the large difference between the solid–plastic transition and melting temperatures resulted in a more significant depression at the eutectic point.

**Figure 4:**
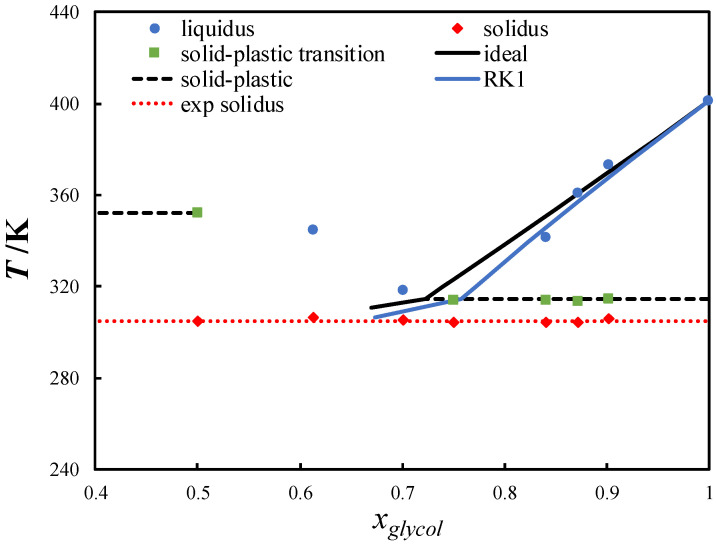
Solid–liquid phase diagram of the choline chloride/neopentyl glycol eutectic system. The black line is the ideal liquidus line of neopentyl glycol, and the blue line was calculated using the Redlich–Kister polynomial equation with one parameter (RK1).

ChCl-based eutectic systems containing diols, such as ChCl/ethylene glycol, have been studied extensively [18,47,48,49,50]. Owing to the low melting temperature and slightly negative deviation from the ideal behavior of most diols, a small depression at the eutectic point is observed in ChCl-based eutectic systems containing diols [18,51,52]. In this work, neopentyl glycol, which possesses a high melting temperature, was mixed with ChCl. Figure 4 shows the SLE phase diagram of the ChCl/neopentyl glycol eutectic system. ChCl is thermally unstable and its melting properties cannot be measured or estimated indirectly [13]. Thus, the ChCl liquidus line could not be calculated. The eutectic temperature was significantly lower than the melting temperature of pure neopentyl glycol (~96 K). Despite the slightly negative deviation from the ideal solution behavior, a large depression at the eutectic point was observed in the system.

### 2.3. Comparison with Other Eutectic Systems

The eutectic systems studied in this work exhibited very low eutectic temperatures compared with the melting temperatures of the pure constituents. Table 1 compares the experimental eutectic temperatures and the differences between the eutectic temperatures and the melting temperatures of plastic or common solid materials ($T_{e}^{exp}$ − *T*_*m*,2_) in various eutectic systems. First, three L-menthol-based eutectic systems containing monocarboxylic acids, namely, L-menthol/pivalic acid, L-menthol/cyclohexane carboxylic acid, and L-menthol/capric acid, are compared. The three eutectic systems are ideal mixtures [35], among which L-menthol/pivalic acid possesses the lowest eutectic temperature and the largest depression at the eutectic point. Thus, when selecting eutectic system constituents from a pool of substances sharing similar chemical natures, PCs can be expected to form deeper eutectics.

Second, three L-menthol-based eutectic systems containing alcohols, i.e., L-menthol/neopentyl alcohol, L-menthol/thymol, and L-menthol/phenol, are compared. L-menthol/thymol and L-menthol/phenol show strong negative deviations from the ideal behavior [53,54]. In contrast, L-menthol/neopentyl alcohol is an ideal eutectic system. As seen in Table 1, the values of ($T_{e}^{exp}$ − *T*_*m*,2_) are considerably small for L-menthol/thymol and L-menthol/phenol. At the same time, L-menthol/neopentyl alcohol shows a significantly low eutectic temperature. Thus, ideal eutectic systems containing PCs can possess lower eutectic temperatures than strongly nonideal eutectic systems.

Third, four eutectic systems containing L-menthol with constituents having high melting temperatures, namely, neopentyl glycol, camphor, borneol, and sobrerol, are compared. As seen in Table 1, the L-menthol/neopentyl glycol, L-menthol/camphor, and L-menthol/borneol eutectic systems show significantly low eutectic temperatures, with ($T_{e}^{exp}$ − *T*_*m*,2_) values of larger than 100 K. In contrast, the eutectic temperature of the L-menthol/sobrerol eutectic system is almost equal to the melting temperature of L-menthol, and its eutectic composition is very close to that of pure L-menthol. This can be attributed to the large difference between the melting temperature of L-menthol and sobrerol [36]. Thus, deep eutectic systems containing constituents that have large differences between their melting temperatures can be formed when the constituent with the high melting temperature is a PC.

ChCl is a PC with a significantly large difference between its solid–plastic transition temperature (351.2 K [13]) and its melting temperature, which should be at least its decomposition temperature (~575 K). This property renders ChCl suitable for forming DESs. The ChCl/urea eutectic system was the first DES that was found to be liquid at room temperature [55]. Urea and neopentyl glycol possess similar melting temperatures; however, urea is not a PC. As seen in Table 1, ChCl/neopentyl glycol shows a similar eutectic temperature and ($T_{e}^{exp}\;$ − *T*_*m*,2_) value to those of ChCl/urea, forming a liquid solution near room temperature. A comparison of a DES containing betaine or sulfamic acid as an HBA instead of ChCl and urea as an HBD with the ChCl/urea eutectic system revealed that the eutectic temperature of the latter is significantly lower than that of the betaine/urea and sulfamic acid/urea eutectic systems. This clearly emphasizes the unique character of ChCl—and PCs in general—for forming DESs. In conclusion, eutectic systems showing significant depressions at the eutectic points were obtained using PCs as one or both constituents. The very low melting entropies and enthalpies of the PCs contributed to the significant depressions in the melting temperatures of the mixtures.

## 3. Materials and Methods

### 3.1. Eutectic Systems

ChCl (≥98%), L-menthol (≥99%), neopentyl alcohol (≥99%), pivalic acid (≥99%), and neopentyl glycol (≥99%) were purchased from Merck (Germany). The ChCl and neopentyl glycol were dried under vacuum (~1 mbar) at 358 and 313 K, respectively, for at least 24 h. The water content of the pure constituents was checked using a Karl Fischer coulometer (Hanna Instrument, Woonsocket, RI, USA) to ensure that it was below 0.1 wt%. The four eutectic systems, namely, L-menthol/neopentyl alcohol, L-menthol/pivalic acid, L-menthol/neopentyl glycol, and ChCl/neopentyl glycol, were prepared by weighing the pure constituents using a balance (precision 0.1 mg, Sartorius, Germany) and mixing them in sealed vials under heat until clear liquids were formed.

### 3.2. DSC

The solid–plastic transition and melting properties of the pure PCs and the SLE data were measured using a DSC instrument (NETZSCH DSC 200 F3, Germany), which was calibrated using adamantane, bismuth, cesium chloride, indium, tin, and zinc as the calibration standards. The standard uncertainties of the sensitivity and temperature measurements were determined to be 0.3% and 0.1 K, respectively.

The DSC crucibles were filled in triplicate with the pure PCs. The samples were melted at a heating rate of 5 K min^−1^, followed by a 5 min isothermal run, and then they were crystallized at a cooling rate of 5 K min^−1^ to a final temperature of 193 K. A second heating run was performed at a rate of 5 K min^−1^. The transition temperature and the transition enthalpy were determined using the second heating run as the onset temperature and the peak area, respectively. The solid–plastic transition and melting temperatures and enthalpies of neopentyl alcohol, pivalic acid, and neopentyl glycol are shown in Table 2.

The eutectic system samples were quenched at 198 K for one hour and annealed at 253 K for one day to facilitate the crystallization. The crystallized samples were ground to a fine powder using a mortar and pestle in a cold room at 253 K. The DSC crucibles were filled with the ground solid in triplicates. The solidus and liquidus temperatures of the samples were measured using DSC at a rate of 5 K min^−1^. The solidus and liquidus temperatures were determined as the onset and peak maximum temperatures of the corresponding peaks, respectively. The experimental SLE data can be found in Tables S1–S4 in the Supplementary Materials.

### 3.3. SLE Modeling

The experimental SLE data confirmed that the system is of the simple eutectic type. The liquidus line of the components showing no solid–plastic transition was calculated as follows:

(1) $$\ln x_{i}^{L}\gamma_{i}^{L}=-\frac{∆h_{m,i}}{RT}\left(1-\frac{T}{T_{m,i}}\right)$$

where $x_{i}^{L}$ and $\gamma_{i}^{L}$ are the mole fraction and activity coefficients of component i in the liquid phase, respectively; *T* is the liquidus temperature; $∆h_{m,i}$ and *T_m,i_* are the melting enthalpy and temperature of component i, respectively; and *R* is the universal gas constant.

For the PCs, the liquidus line was calculated as follows [60]:

(2) $$\ln x_{i}^{L}\gamma_{i}^{L}=-\frac{∆h_{m,i}}{RT}\left(1-\frac{T}{T_{m,i}}\right)-\frac{∆h_{tr,i}}{RT}\left(1-\frac{T}{T_{tr,i}}\right)$$

where $∆h_{tr,i}$ and *T_tr,i_* are the solid–plastic transition enthalpy and temperature of component i, respectively.

The SLE phase diagram of the L-menthol-based eutectic systems was calculated assuming the ideal solution model, i.e., $\gamma_{i}^{L}=1$, using Equations (1) and (2) for the L-menthol and the PCs, respectively. The solid–plastic transition and melting properties of the PCs measured in this work (Table 2) were used, and those of L-menthol were taken from the literature (*T_m,i_* = 314.6 K and Δ*h_m,i_* = 13.74 kJ mol^−1^ [35]).

The ChCl/neopentyl glycol eutectic system showed a negative deviation from the ideal behavior. The activity coefficients of neopentyl glycol in the ChCl/neopentyl glycol eutectic system were calculated using the following Redlich–Kister polynomial equation, with one parameter [61]:

(3) $$\ln\gamma_{i}^{L}=\frac{A}{RT}\left(1-x_{i}\right)$$

The binary interaction parameter A was fitted to the neopentyl glycol experimental liquidus data ($T_{i}^{exp}$), minimizing the following objective function:

(4) $$F\left(T\right)=\overset{n}{\underset{i=1}{\sum }}{\left(\frac{{\left(T_{i}^{exp}-T_{i}^{cal}\right)}^{2}}{n}\right)}^{\frac{1}{2}}$$

where *n* is the number of data points. The calculated binary interaction parameter A was −2.9192 kJ mol^−1^.

## 4. Conclusions

This work demonstrates the formation of eutectic systems with low eutectic temperatures using PCs as constituents. Three PCs having chemical natures resembling common HBDs used in the DES literature, namely, monocarboxylic acids, alcohols, and diols, were selected in this work. The solid–plastic transition and melting properties of the three PCs were measured using DSC. The SLE phase diagrams of four eutectic systems containing the three PCs and L-menthol or ChCl were determined via DSC and modeled using the ideal solution model and Redlich–Kister polynomial equation (with one parameter).

The SLE phase diagrams reported in this work showed that PCs can form eutectic systems with large depressions at the eutectic points relative to the melting temperatures of the pure constituents. Despite having melting temperatures significantly higher than those of their structural isomers, the PCs possess very low melting enthalpies and entropies, increasing the slopes of the liquidus lines of the pure constituents above the solid–plastic transition temperatures and resulting in low eutectic temperatures. However, because the solid–plastic transition enthalpies are larger than the melting enthalpies of the PCs, the slopes of the liquidus lines below the solid–plastic transition temperatures become less steep. Hence, PCs with large differences between the solid–plastic transition temperatures and the melting temperatures were found to form deeper eutectics.

Despite the nearly ideal behavior observed in the studied systems, the eutectic temperature depressions were comparable or even larger than those observed in strongly nonideal eutectic systems containing common crystalline solids. For practical applications, it is more important to have a DES that is liquid and lowly viscous at the operating conditions independent of the nonideality of the system. Thus, ideal eutectic systems containing PCs could be promising green solvents for various applications.

## Figures and Tables

**Table 1:** A comparison between the melting temperatures of pure constituents (*T_m_*), the experimental eutectic temperatures ($T_{e}^{exp}$), and the depressions at the eutectic points relative to the melting temperatures ($T_{e}^{exp}$ − *T*_*m*,2_) of the pure constituents of eutectic systems studied in this work and found in the literature.

Component 1	Component 2	*T_m,2_*/K	$T_{e}^{exp}/K$	$(T_{e}^{exp}\;-T_{m,2})/K$
L-menthol	Pivalic acid ^a,b^	308.7	260.6	−48.1
	Cyclohexane carboxylic acid [35]	299.4	265.0	−34.4
	Capric acid [35]	303.9	279.0	−24.9
	Neopentyl alcohol ^a,b^	328.9	259.2 ^c^	−69.7
	Thymol [53]	322.7	271.7	−51.0
	Phenol [54]	313.9	261.3	−52.6
	Neopentyl glycol ^a,b^	401.2	291.8	−109.4
	Camphor ^a^ [32]	450.4	275.7	−174.7
	Borneol ^a^ [32]	480.6	286.7	−193.9
	Sobrerol [32]	420.2	– ^d^	–
Choline chloride ^a^	Neopentyl glycol ^a,b^	401.2	305.1	−96.1
	Urea [55]	405.2	297.7	−107.5
Betaine	Urea [56]	405.2	359.3	−45.9
Sulfamic acid	Urea [57]	405.2	351.1	−54.1

Note: ^a^ plastic crystalline material; ^b^ measured in this work; ^c^ ideal eutectic temperature; and ^d^ the eutectic composition is near pure L-menthol.

**Table 2:** Transition temperatures (*T_tr_*) and enthalpies (Δ*h_tr_*) and melting temperatures (*T_m_*) and enthalpies (Δ*h_m_*) of the studied plastic crystalline materials.

Compound	*T_tr_*/K		Δ*h_tr_*/kJ mol^−1^		*T_m_*/K		Δ*h_m_*/kJ mol^−1^	
	This work	Literature	This work	Literature	This work	Literature	This work	Literature
Neopentyl alcohol	235.9 ± 0.1	242.1 ^a^	4.46 ± 0.06	4.6 ^a^	328.9 ± 1.2	328.1 ^a^	4.01 ± 0.03	3.5 ^a^
Pivalic acid	279.7 ± 0.1	278.3 ^b^	7.99 ± 0.32	8.18 ^b^	308.7 ± 0.2	309.1 ^b^	2.15 ± 0.13	2.27 ^b^
Neopentyl glycol	314.7 ± 0.1	315.2 ^a^	12.86 ± 0.20	12.8 ^a^	401.2 ± 0.1	402.5 ^a^	4.16 ± 0.06	4.3 ^a^

Note: ^a^ data were taken from Granzow [58], and ^b^ data were taken from Singh and Glicksman [59].

## Data Availability

All data supporting the reported results can be found within the manuscript and the Supplementary Materials.

## Supplementary Materials

The following supporting information can be downloaded at: https://www.mdpi.com/article/10.3390/molecules27196210/s1, Table S1. Solid–liquid equilibria data of the L-menthol/neopentyl alcohol eutectic system. Table S2. Solid–liquid equilibria data of the L-menthol/pivalic acid eutectic system. Table S3. Solid–liquid equilibria data of the L-menthol/neopentyl glycol eutectic system. Table S4. Solid–liquid equilibria data of the ChCl/neopentyl alcohol eutectic system.

## Sample Availability

Samples of the studied compounds and mixtures are unavailable.
