# Supplementary material for: Design of Deep Eutectic Systems: Plastic Crystalline Materials as Constituents
Source: Molecules. 2022 Sep 21;27(19):6210. doi: 10.3390/molecules27196210 (PMC9573734; doi:10.3390/molecules27196210)
Supplement: Supplementary file 1 [file molecules-27-06210-s001.zip › molecules-1911453-supplementary.pdf]

Supplementary Materials

Design of Deep Eutectic Systems: Plastic Crystalline Materials as Constituents

Ahmad Alhadid [1,*], Sahar Nasrallah [1], Liudmila Mokrushina [2] and Mirjana Minceva [1]

[1] Biothermodynamics, TUM School of Life Sciences, Technical University of Munich (TUM), Maximus-von-Imhof-Forum 2, 85354 Freising, Germany

[2] Separation Science & Technology, Friedrich-Alexander-Universität Erlangen-Nürnberg (FAU), Egerlandstr. 3, 91058 Erlangen, Germany

* Correspondence: ahmad.alhadid@tum.de

Table S1. Solid–liquid equilibria data of the L-menthol/neopentyl alcohol eutectic system.

Neopentyl alcohol mole fraction	Solidus temperature	Liquidus temperature
0.246	–	299.1 ± 1.3
0.136	–	306.7 ± 0.1

uncertainties are the standard deviation of three measurements

Table S2. Solid–liquid equilibria data of the L-menthol/pivalic acid eutectic system.

Pivalic acid mole fraction	Solidus temperature	Liquidus temperature
0.898	260.7 ± 0.5	279.2 ± 0.7
0.844	261.3 ± 0.2	276.0 ± 0.1
0.751	259.9 ± 0.1	269.1 ± 0.1
0.602	261.9 ± 0.1	–
0.551	261.9 ± 0.1	–
0.392	260.1 ± 0.1	–
0.355	259.2 ± 0.7	284.1 ± 0.1
0.310	259.7 ± 0.1	288.7 ± 0.5
0.198	260.1 ± 0.5	300.2 ± 0.2
0.105	261.6 ± 0.1	309.3 ± 0.1

uncertainties are the standard deviation of three measurements

Table S3. Solid–liquid equilibria data of the L-menthol/neopentyl glycol eutectic system.

Neopentyl glycol mole fraction	Solid–solid transition	Solidus temperature	Liquidus temperature
0.900	315.0 ± 0.1	290.3 ± 0.3	378.7 ± 1.7
0.853	314.9 ± 0.1	290.9 ± 0.1	367.7 ± 0.1
0.798	314.9 ± 0.1	291.5 ± 0.1	349.5 ± 2.5
0.600		292.5 ± 0.1	310.4 ± 1.0
0.491		292.9 ± 0.1	303.3 ± 0.1
0.456		292.7 ± 0.2	–
0.395		292.3 ± 0.4	–
0.251		292.3 ± 0.1	298.7 ± 0.1
0.209		291.9 ± 0.1	302.3 ± 0.1
0.142		291.2 ± 0.2	306.9 ± 0.1
0.098		291.7 ± 0.1	309.5 ± 0.1

uncertainties are the standard deviation of three measurements

Table S4. Solid–liquid equilibria data of the choline chloride/neopentyl glycol eutectic system.

Neopentyl glycol mole fraction	Solid–solid transition	Solidus temperature	Liquidus temperature
0.902	314.6 ± 0.1	305.9 ± 0.1	373.2 ± 0.5
0.872	313.3 ± 0.1	304.4 ± 0.7	360.9 ± 1.1
0.841	314.1 ± 0.2	304.1 ± 0.1	341.6 ± 0.1
0.750	314.3 ± 0.2	304.3 ± 1.3	–
0.701	–	305.3 ± 0.5	318.5 ± 0.1
0.613	–	306.7 ± 0.7	344.5 ± 0.1
0.500	352.3 ± 0.2	305.1 ± 0.1	–

uncertainties are the standard deviation of three measurements
